# Pharmacometric estimation methods for aggregate data, including data simulated from other pharmacometric models

**DOI:** 10.1007/s10928-021-09760-1

**Published:** 2021-06-22

**Authors:** Pyry Antti Juhana Välitalo

**Affiliations:** 1 School of Pharmacy, University of Eastern Finland, Yliopistonranta 1 C, 70210 Kuopio, Finland; 2 Finnish Medicines Agency, Microkatu 1, 70210 Kuopio, Finland

**Keywords:** Pharmacometrics, Population pharmacokinetics, Aggregate data, Model-based meta-analysis

## Abstract

Lack of data is an obvious limitation to what can be modelled. However, aggregate data in the form of means and possibly (co)variances, as well as previously published pharmacometric models, are often available. Being able to use all available data is desirable, and therefore this paper will outline several methods for using aggregate data as the basis of parameter estimation. The presented methods can be used for estimation of parameters from aggregate data, and as a computationally efficient alternative for the stochastic simulation and estimation procedure. They also allow for population PK/PD optimal design in the case when the data-generating model is different from the data-analytic model, a scenario for which no solutions have previously been available. Mathematical analysis and computational results confirm that the aggregate-data FO algorithm converges to the same estimates as the individual-data FO and yields near-identical standard errors when used in optimal design. The aggregate-data MC algorithm will asymptotically converge to the exactly correct parameter estimates if the data-generating model is the same as the data-analytic model. The performance of the aggregate-data methods were also compared to stochastic simulations and estimations (SSEs) when the data-generating model is different from the data-analytic model. The aggregate-data FO optimal design correctly predicted the sampling distributions of 200 models fitted to simulated datasets with the individual-data FO method.

**Graphic abstract:**

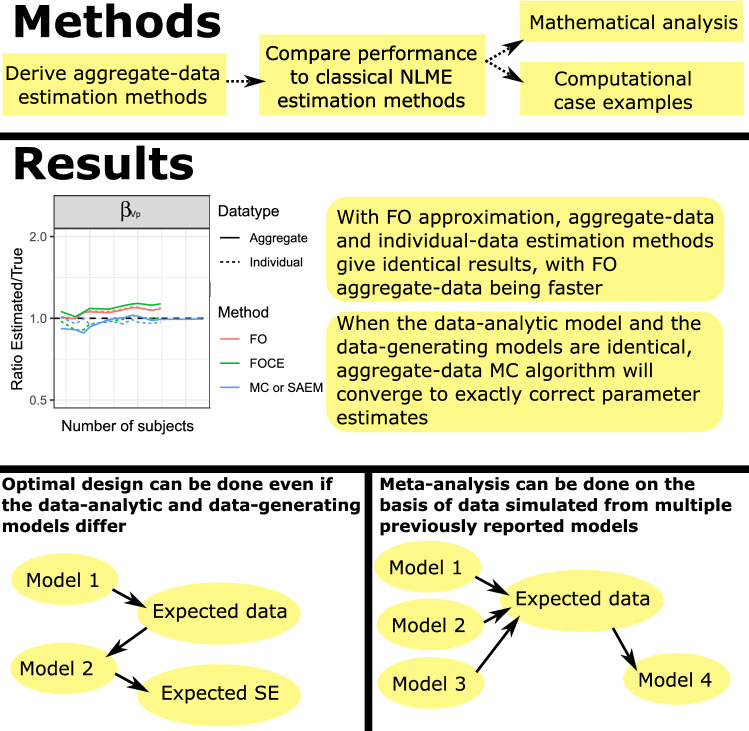

**Supplementary Information:**

The online version contains supplementary material available at 10.1007/s10928-021-09760-1.

## Introduction

Population pharmacokinetic estimation by nonlinear mixed-effects models was introduced by Sheiner and Beal [1–4]. In their seminal work, the authors outlined how the nonlinear mixed-effects model can be linearized around the expected values of the random effects. In practice, this enabled sparse data from individuals to be included in mathematical models of drug concentration and effect. The original estimation algorithm was named the first-order method, or the FO method for short. Several other, more accurate, estimation methods were later introduced. These estimation methods require individual-level data from subjects.

In this paper, the term “aggregate data” refers to data such as mean observed concentrations and the variance–covariance matrix of the observed concentrations. The need to analyze aggregate data may arise e.g. when performing a model-based meta-analysis. A recent example of model-based meta-analysis was published by Weber et al. [5]. In their example, a Bayesian approach for the joint analysis of individual-level data and aggregate mean data was developed. The mean aggregate data, extracted from literature, were included in the calculation of overall likelihood as data points. The observed means were contrasted with model-predicted means and covariances of simulated datapoints. The authors discussed that the mean aggregate data were generally informative for fixed-effect parameters but not for random-effects parameters. As such, the mean data alone are not enough to inform pharmacometric models. An estimation method which would allow estimation of all parameters including fixed effects, random effects and residual variability on the basis of aggregate data alone would be desirable.

Nonlinear mixed-effects models can be complex, and thus it can be complex to design experiments that aim to utilize these models. Therefore, tools that help in designing these experiments are relevant. Optimal design refers to calculation of expected Fisher Information Matrix (FIM), given a study design and some assumed model. Calculation of expected FIM for nonlinear mixed-effects models was originally reported by Mentre and coworkers using the first-order linearization [6]. One currently present limitation of optimal design is that the same model must be assumed for both data generation and data analysis.

The purpose of this manuscript is to outline an approach for fitting full pharmacometric models to aggregate data consisting of mean vector and variance–covariance matrix. The maximum likelihood estimators for aggregate data are presented. It is shown that the mean vector and variance–covariance matrix can be simulated from a priori defined models. This enables the model-based meta-analysis of data which are extracted from multiple previously reported models. It also allows optimal design in the case when the data-analytic and the data-generating models differ.

## Theoretical

### Definitions and notation

This manuscript uses a column vector notation and “log” refers to natural logarithm. The word “design” refers to the independent variables of the data. Usually, the most obvious design aspect is the PK/PD sampling schedule.

A design for a multi-response mixed-effects model is composed of *N* subjects, each with an associated elementary design ${\varvec{\xi}}_{i} \left( {i = 1,...,N} \right)$; hence, a design for a population of *N* subjects can be described as follows:

1 $${\varvec{\varXi}}= \left( {{\varvec{\xi}}_{1} , \ldots ,{\varvec{\xi}}_{N} } \right)$$

Each elementary design ${\varvec{\xi}_{i}}$ can be further divided into subdesigns:

2 $${\varvec{\xi}}_{i} = \left( {\xi_{i1} , \ldots ,\xi_{iK} } \right)$$

With ${\varvec{\xi}_{ik}}$, $k = 1,...,K$ being the design associated with the *k*th response such as drug concentration or drug effect. In this manuscript dealing with aggregate data, an assumption is made that the elementary designs can also be grouped across individuals. However, the framework also allows elementary designs unique to an individual.

The individual response $y_{ik}$ is modelled as follows:

3 $$y_{ik} = f_{k} \left( {{\varvec{\theta}}_{i} ,\xi_{ik} } \right) + h_{k} \left( {{\varvec{\theta}}_{i} ,\xi_{ik} ,\varepsilon_{ik} } \right)$$

where *f*_*k*_(.) is the structural model for the *k*th response, ${\varvec{\theta}}_{i}$ is the *i*th subject’s parameter vector, *h*_*k*_(.) is the residual error model for response *k*, (often additive, proportional or a combination of additive and proportional), and $\varepsilon_{ik}$ is the residual error for response *k* in subject *i*. The general prediction and residual error functions for all responses are denoted by *f*() and *h*(), respectively. The residual error $\varepsilon_{ik}$ are distributed with a mean of zero and additive and proportional variance terms as elements of ${\varvec{\Sigma}}$. A matrix ***Y*** of responses is defined as:

4 $${\varvec{Y}} = \left[ {\begin{array}{*{20}c} {y_{11} } & {y_{12} } & \ldots & {y_{1K} } \\ {y_{21} } & {y_{22} } & {} & {} \\ \vdots & {} & \ddots & {} \\ {y_{N1} } & {} & {} & {y_{NK} } \\ \end{array} } \right]$$

The individual parameter vector ${\varvec{\theta}}_{i}$, with parameter(s) that might be shared between responses, is described as follows:

5 $${\varvec{\theta}}_{i} = g\left( {{\varvec{\beta}},{\varvec{b}}_{i} } \right)$$

where ${\varvec{\beta}}$ is the vector of fixed effects parameters, or typical subject parameter and ***b***_*i*_ is the vector of *v* random effects for subject *i*. The random effects ***b***_*i*_ are assumed to be normally distributed with a mean of zero and a covariance matrix ${\varvec{\varOmega}}$ of size $v \times v$ with off-diagonal elements either as parameters to be estimated, or fixed to zero. The vector of population parameters is thus defined as follows:

6 $${\varvec{\varPsi}}= \left[ {{\varvec{\beta}},{\varvec{vec}}\left({\varvec{\varOmega}}\right),{\varvec{vec}}\left({\varvec{\Sigma}}\right)} \right] = \left[ {{\varvec{\beta}},\omega_{1}^{2} ,\omega_{12} ,\omega_{2}^{2} , \ldots ,\omega_{v}^{2} ,\sigma_{1}^{2} ,\sigma_{2}^{2} } \right]$$

where ${\varvec{vec}}\left( . \right)$ refers to vectorization operation.

The aggregate mean response vector $\overline{y}_{{\text{k}}}$ at subdesign $\xi_{k}$ and the aggregate (co)variance response $V_{kl}$ between subdesigns ${\varvec{\xi}}_{*k}$ and ${\varvec{\xi}}_{*l}$ are defined as

7 $$\overline{y}_{{\text{k}}} = \frac{1}{{\text{N}}}\mathop \sum \limits_{i = 1}^{n} y_{ik}$$

8 $$V_{kl} = \frac{1}{{\text{N}}}\mathop \sum \limits_{i = 1}^{n} \left( {y_{ik} - \overline{y}_{k} } \right)\left( {y_{il} - \overline{y}_{l} } \right)$$

The mean vector $\overline{\user2{y}}$ consists of $\left( {\overline{y}_{1} ,\overline{y}_{2} ,...,\overline{y}_{K} } \right)$ and the variance–covariance matrix ***V*** of the observed data is a K-K matrix, the elements of which correspond to the elements $V_{kl}$.

### Parameter estimation when individual-level data are available

This subsection presents already known and published results about parameter estimation when individual-level data are available. It is included as an introductory, relevant background information.

The likelihood $L({\varvec{y}}_{i} |{\varvec{\varPsi}})$ of observed data ${\varvec{y}}_{i}$ for individual *i* given parameters ${\varvec{\varPsi}}$, is defined as

9 $$L\left( {{\varvec{y}}_{i} |{\varvec{\varPsi}}} \right) = \smallint l\left( {{\varvec{y}}_{i} {|}{\varvec{\theta}}_{i} ,{\varvec{\xi}}_{i} } \right)p\left( {{\varvec{\theta}}_{i} {|}{\varvec{\varPsi}}} \right)d{\varvec{\theta}}_{i}$$

where $l({\varvec{y}}_{i} |{\varvec{\theta}}_{i} ,{\varvec{\xi}}_{i} )$ is the conditional likelihood of ${\varvec{y}}_{i}$ given individual parameters ${\varvec{\theta}}_{i}$ and design factors ${\varvec{\xi}}_{i}$.

The overall likelihood function $L({\varvec{Y}}|{\varvec{\varPsi}})$ for the data is the product of individual likelihood functions, or mathematically $L({\varvec{Y}}|{\varvec{\varPsi}}) = \prod L({\varvec{y}}_{i} |{\varvec{\varPsi}})$. To avoid floating point errors associated with very high or low numerical values, it is common to maximize a log-likelihood function, equal to the sum of individual log-likelihoods.

In FO and FOCE approximations, the individual data are treated as multi-variate normally distributed. The log-likelihood can then be expressed as

10 $$\begin{gathered} \log \left( {L\left( {{\varvec{y}}_{i} \left|{\varvec{\varPsi}}\right.} \right)} \right) = - \frac{1}{2}\left( {{\varvec{y}}_{res,i}^{T} \tilde{\user2{V}}_{i}^{ - 1} {\varvec{y}}_{res,i} + \log \left| {\tilde{\user2{V}}_{i} } \right|} \right) \hfill \\ \,\,\,\,\,\,\,\,\,\,\,\,\,\,\,\,\,\,\,\,\,\,\,\,\,\,\,\,\,\,\,\,\,\,\, = - \frac{1}{2}\left( {tr\left( {{\varvec{R}}_{i} \tilde{\user2{V}}_{i}^{ - 1} } \right) + \log \left| {\tilde{\user2{V}}_{i} } \right|} \right) \hfill \\ \end{gathered}$$

where ${\varvec{y}}_{res,i}$ is the vector of residuals, ${\varvec{R}}_{i}$ is the outer product of the residuals ${\varvec{R}}_{i} = {\varvec{y}}_{res,i}^{T} \times {\varvec{y}}_{res,i}$ and $\tilde{\user2{V}}_{i}$ is the individual predicted variance–covariance matrix. To clarify the above expressions, we note that

11 $${\varvec{y}}_{res,i}^{T} \tilde{\user2{V}}_{i}^{ - 1} {\varvec{y}}_{res,i} = \mathop \sum \limits_{k} y_{res,ik} \left( {\mathop \sum \limits_{l} y_{res,il} \left( {\tilde{\user2{V}}_{i}^{ - 1} } \right)_{kl} } \right) = tr\left( {{\varvec{R}}_{i} \tilde{\user2{V}}_{i}^{ - 1} } \right)$$

With nonlinear mixed-effects models, there is no closed-form solution to $L\left( {{\varvec{y}}_{i} } \right)$. Therefore, various approximations have been developed. The FO approximation linearizes the model around the expected average value of the random effect at zero, and FOCE approximation linearizes the model around the conditional maximum a posteriori estimates of ${\varvec{b}}_{i}$.

12 $${\varvec{y}}_{res,FOCE,i} = {\varvec{y}}_{i} - \left( {f\left( {\tilde{\user2{\varvec\theta }}_{i} ,{\varvec{\xi}}_{i} } \right) - \frac{{\delta f\left( {\tilde{\user2{\varvec\theta }}_{i} ,{\varvec{\xi}}_{i} } \right)}}{{\delta {\varvec{b}}_{i} }}\tilde{\user2{b}}_{i} } \right)$$

13 $$\tilde{\user2{V}}_{FOCE,i} = \left( {\frac{{\delta f\left( {\tilde{\user2{\varvec\theta }}_{i} ,{\varvec{\xi}_{i}} } \right)}}{{\delta {\varvec{b}}_{i} }}} \right){\varvec{\varOmega}}\left( {\frac{{\delta f\left( {\tilde{\user2{\varvec\theta }}_{i} ,{\varvec{\xi}_{i}} } \right)}}{{\delta {\varvec{b}}_{i} }}} \right)^{T} + diag\left( {\left( {\frac{{\delta h\left( {\tilde{\user2{\varvec\theta }}_{i} ,{\varvec{\xi}}_{i} ,\varepsilon_{i} } \right)}}{{\delta \varepsilon_{i} }}} \right){{\varvec{\Sigma}}}\left( {\frac{{\delta h\left( {\tilde{\user2{\varvec\theta }}_{i} ,{\varvec{\xi}}_{i} , \varepsilon_{i} } \right)}}{{\delta\varepsilon_{i} }}} \right)^{T} } \right)$$

In the above equations, expression $\frac{{\delta f\left( {{\varvec{\theta}}_{i} ,{\varvec{\xi}}_{i} } \right)}}{{\delta {\varvec{b}}_{i} }}$ is the Jacobian matrix of model predictions with regard to random effects, with *K* number of rows and *v* number of columns, evaluated at $\tilde{\user2{b}}_{i}$. Similarly, expression $\frac{{\delta h\left( {{\varvec{\theta}}_{i} ,{\varvec{\xi}_{i}} ,\varvec{\epsilon}_{i} = 0} \right)}}{{\delta \varvec{\epsilon}_{i} }}$ is the Jacobian matrix of residual variability with regard to the residual variability terms, evaluated at $\varvec{\epsilon}_{i} = \vec{0}$, with *K* number of rows and number of columns equal to the number of residual variability terms. In FOCE method, $\tilde{\user2{b}}_{i}$ is the vector of maximum a posteriori estimates of the random effects for individual *i*, given individual data ${\varvec{y}}_{i}$ and population parameters ${\varvec{\varPsi}}$. Similarly, $\tilde{\user2{\varvec\theta }}_{i}$ is the vector of individual parameters that result from substituting $\tilde{\user2{b}}_{i}$ to equation 5. In FO method, $\tilde{\user2{b}}_{i}$ is a vector of zeros, therefore the above equations also cover FO approximation as a special case.

It is relevant to note that maximizing the log-likelihood with regard to the FO and FOCE approximations is not guaranteed to lead to exactly correct parameter estimates, even if the amount of subjects in dataset would approach infinity. The expected mismatch between the true values and the parameter estimates becomes more pronounced as either the variance of random effects increases, or as the number of data points per individual decreases. Newer, EM-based estimation algorithms such as the importance sampling EM algorithm [7] and the SAEM algorithm [8–10] do not have this problem. While these EM-based algorithms are still “approximations” because of Monte-Carlo sampling, they will converge to the exact maximum likelihood parameter estimates, given a sufficiently high number of iterations and Monte-Carlo samples of random effects. For reasons of conciseness, the mathematical details of importance sampling and SAEM estimation algorithms will not be covered here.

### Estimation based on aggregate data

For *N* individuals sharing the same study design, the FO log-likelihood expression introduced in equation 10 can be simplified to equation 14. For detailed steps, please refer to Appendix 1.

14 $$\log \left( {L\left( {\overline{\user2{y}},{\varvec{V}}{|}{\varvec{\varPsi}}} \right)} \right) = - \frac{N}{2}\left( {tr\left( {{\varvec{V}} \cdot \tilde{\user2{V}}^{ - 1} } \right) + \left( {\overline{\user2{y}} - \tilde{\user2{y}}} \right)^{T} \tilde{\user2{V}}^{ - 1} \left( {\overline{\user2{y}} - \tilde{\user2{y}}} \right) + {\text{log}}\left| \tilde{\user2{V}} \right|} \right)$$

In the above equation, $\tilde{\user2{y}}$ is the vector of mean model predictions. This log-likelihood expression is highly similar to the expression used for fitting structural equation models to variance terms. As shown by Jöreskog [11], the observed variance–covariance matrix of observations is Wishart distributed and therefore the log-likelihood expression for $\tilde{\user2{V}}_{i}$ is identical to equation 14 with the $\left( {\overline{\user2{y}} - \tilde{\user2{y}}} \right)^{T} \tilde{\user2{V}}^{ - 1} \left( {\overline{\user2{y}} - \tilde{\user2{y}}} \right)$ term omitted.

The proposed expressions for the log-likelihood of aggregate data are highly similar to the expression for the log-likelihood of individual data. Both expressions involve taking the log-determinant of the predicted variance–covariance matrix, the only difference is in use of $\tilde{\user2{V}}$ versus $\tilde{\user2{V}}_{i}$. Further, both expressions involve taking the trace of residual variance–covariance matrix (either ${\varvec{V}}_{i}$ or **V**) multiplied by the inverse of the predicted variance–covariance matrix. A major difference between the two methods is that the individual likelihood $L({\varvec{y}}_{i} |{\varvec{\varPsi}})$ is calculated by integrating over the random effect values while considering the individual data ${\varvec{y}}_{i}$. For the analysis of aggregate data, such a thing is not possible, and instead it is only possible to integrate over the (unknown) random effect values while considering the aggregate data; the mean vector and variance–covariance matrix of observations.

Although equation 14 was transformed from the log-likelihood of FO method, it applies generally to aggregate data consisting of means and variance–covariance matrices (please see Appendix 2).

### FO, FOCE and Monte–Carlo approximations of the predicted aggregate data

In this manuscript, three methods for integrating over the random effect values ${\varvec{b}}_{i}$ will be proposed: The already mentioned aggregate-data FO method, the aggregate-data FOCE method, and the aggregate-data MC method. These three methods are fed into computer optimization algorithms.

The FO aggregate method is identical to the FO method for non-aggregate data. For the FOCE aggregate method, it is not possible to estimate maximum a posteriori values of $\tilde{\user2{b}}_{i}$ because individual data are not available. Therefore, the FOCE aggregate method consists of Monte Carlo integration over a set of quasi-randomly sampled values of $\tilde{\user2{b}}_{i}$, similar but not identical to the optimal design FOCE-like approximation described by Retout and Mentré [12].

15 $$\tilde{\user2{y}}_{FOCE} = \frac{1}{{N_{sim} }}\mathop \sum \limits_{i}^{{N_{sim} }} \left( {f\left( {{\varvec{\theta}}_{i} ,{\varvec{\xi}}_{i} } \right) - \frac{{\delta f\left( {{\varvec{\theta}}_{i} ,{\varvec{\xi}}_{i} } \right)}}{{\delta {\varvec{b}}_{i,sim} }}{\varvec{b}}_{i,sim} } \right)$$

16 $$\tilde{\user2{V}}_{FOCE} = \frac{1}{{N_{sim} }}\mathop \sum \limits_{i}^{{N_{sim} }} \left( {\frac{{\delta f\left( {{\varvec{\theta}}_{i} ,{\varvec{\xi}_{i}} } \right)}}{{\delta {\varvec{b}}_{i,sim} }}} \right){\varvec{\varOmega}}\left( {\frac{{\delta f\left( {{\varvec{\theta}}_{i} ,{\varvec{\xi}_{i}} } \right)}}{{\delta {\varvec{b}}_{i,sim} }}} \right)^{T} + diag\left( {\left( {\frac{{\delta h\left( {{\varvec{\theta}}_{i} ,{\varvec{\xi}_{i}} ,\varepsilon_{i} } \right)}}{{\delta \varepsilon_{i} }}} \right){\varvec{\varSigma}}\left( {\frac{{\delta h\left( {{\varvec{\theta}}_{i} ,{\varvec{\xi}_{i}} ,\varepsilon_{i} } \right)}}{{\delta \varepsilon_{i} }}} \right)^{T} } \right)$$

where ${\varvec{b}}_{i,sim}$ is one simulated vector of random effect values out of the total of $N_{sim}$ simulated random effect vectors. The differences between equations 15–16 and the FOCE-like approximation described by Retout and Mentré [12] are explored in the Discussion: Limitations subsection.

At this point it may be observed that numerical integration could be done directly on the raw simulated data instead of doing numerical integration over a first-order approximation. For this reason, the aggregate-data MC method is introduced.

17 $$\tilde{\user2{y}}_{MC} = \frac{1}{{N_{sim} }}\left( {\vec{1}_{N}^{T} {\varvec{Y}}_{sim} } \right)^{T}$$

18 $$\tilde{\user2{V}}_{MC} = \frac{1}{{N_{sim} }}\left( {{\varvec{Y}}_{sim} - \vec{1}_{N}^{T} \otimes \tilde{\user2{y}}_{MC} } \right)^{T} \left( {{\varvec{Y}}_{sim} - \vec{1}_{N}^{T} \otimes \tilde{\user2{y}}_{MC} } \right) + \frac{1}{{N_{sim} }}\mathop \sum \limits_{i}^{{N_{sim} }} diag\left( {\left( {\frac{{\delta h\left( {{\varvec{\theta}}_{i} ,{\varvec{\xi}}_{i} ,\varepsilon_{i} = 0} \right)}}{{\delta \varepsilon_{i} }}} \right){\varvec{\varSigma}}\left( {\frac{{\delta h\left( {{\varvec{\theta}}_{i} ,{\varvec{\xi}}_{i} ,\varepsilon_{i} = 0} \right)}}{{\delta \varepsilon_{i} }}} \right)^{T} } \right)$$

where $\vec{1}_{N}^{T}$ is a vector of ones of length $N_{sim}$, symbol $\otimes$ refers to outer product, and ${\varvec{Y}}_{sim}$ is the $N_{sim} \times K$ matrix of simulated responses, based on function *f* and a set of Sobol-sampled values of ${\varvec{b}}_{sim}$. This method allows interaction between ***b*** and $\varvec\varepsilon$ values. It is noted that if residual error distribution is symmetric (additive, proportional or additive + proportional), then the MC aggregate method, at sufficiently high $N_{sim}$, will produce exactly correct predictions of mean vector and variance–covariance matrix. Therefore, if the above conditions are fulfilled, the MC aggregate method will converge to the exact maximum likelihood estimates of the aggregate data.

### Applications

The aggregate data log-likelihood expressions can be directly applied in estimation, optimal design and as a replacement to stochastic simulation and estimation.

The expressions can be readily used to fit models to observed aggregate data, possibly side to side with individual-level data. For FO method, the aggregate-data estimation is guaranteed to give results identical to individual-data estimation (Appendix 1). For the aggregate-data MC method, there is a guarantee that given a sufficiently high $N_{sim}$ and sufficiently high number of subjects in the dataset, the parameter estimates will converge to the same ones as with individual-data SAEM algorithm, if the data-generating model and the data-analytic model are the same. However, if the data-generating model and the data-analytic model are different, then individual-data SAEM algorithm will allow the random effects distributions to differ from normality to some degree; the aggregate-data MC algorithm is not able to do this. Therefore, when data-generating and data-analytic models are different, the aggregate-data MC results are likely to differ from individual-data SAEM results. Finally, for FOCE algorithm, there is no guarantee that the aggregate-data and individual-data methods give identical results. This is explored in detail in section Discussion: Limitations.

The newly developed expressions are also readily applicable to optimal design. One can simply simulate the expected aggregate data based on equations 15–18 and take the numerical Hessian of log-likelihood for the simulated aggregate data with respect to model parameters. Appendix 3 proves that taking the Hessian of the aggregate data log-likelihood directly results in the published optimal design expressions [12] for the FO approximation of population Fisher Information Matrix.

Finally, the newly developed expressions for aggregate data log-likelihood can be used as a replacement to procedures known as stochastic simulation and estimation (SSE). In these procedures, multiple datasets are simulated under the evaluated design, one or more models are fitted to each of the simulated datasets, and summaries of the parameter estimates across all fitted models are calculated. The newly developed expressions can be used to first simulate expected aggregate data from the data-generating model according to equations 15–18, and then to fit the data-analytic model to the simulated aggregate data. This needs to be done only once with sufficiently high $N_{sim}$, and not for multiple simulated datasets.

## Methods

Four case studies are presented. The first one compares the aggregate data log-likelihood to previously published log-likelihood calculations in a paper containing the derivation of NONMEM estimation methods [13]. The second case example examines the estimation properties of the aggregate data estimation methods and compares them to the classical estimation methods based on individual data. A simulated dataset with an increasing number of simulated subjects is used, and convergence to the correct parameter values is monitored. Finally, the third and fourth case examples examine the potential of the newly derived expressions to be used in optimal design, and as a replacement for stochastic simulations and estimations.

We emphasize that these simulation case examples function as sanity checks to demonstrate some specific features of the aggregate data estimation methods. The case examples alone do not prove anything. The actual proofs regarding the aggregate data estimation methods are mathematical.

### Case 1: log-likelihood value comparison

This case example shows that the aggregate-data FO approximation results in exactly identical objective function value as the individual-data FO approximation. For this case example, the model and dataset described and used by Wang [13] were utilized due to their public availability. The data table can be observed in the original publication, and will not be repeated here. As described in the original publication [13], the model for *k*th measurement of *i*th subject is specified as:

$$y_{ik} = 10 \cdot exp\left( { - \beta_{1} \times exp\left( {b_{i,1} } \right) \cdot t_{ik} } \right) + \varepsilon_{ik}$$

where the distribution of $\varepsilon$ depends on the residual error model. The model objective function was calculated at the same parameter estimates as used in the original publication, namely $\beta_{1} = 0.5$, $\omega_{1}^{2} = 0.04$, $\sigma_{1}^{2} = 0.1$. Objective function values with additive and proportional residual error models were evaluated. The FO and FOCE algorithms were used both for individual-data and aggregate-data estimation.

### Case 2: parameter estimation accuracy

This case example demonstrates that the aggregate-data MC approximation converges to the correct parameter estimates when the size of the dataset is sufficiently high. It also further demonstrates that the individual-data and aggregate-data FO approximations result in identical parameter estimates. A two-compartment mammillary model with first-order absorption and elimination is used. Parameter values of 5 L/h clearance, 10 L central volume of distribution, 30 L peripheral volume of distribution, 10 L/h inter-compartmental clearance and 1/h absorption rate constant are used for fixed effects. Log-normally distributed inter-individual variability with log-standard deviation of 0.3 is used for all parameters. No correlations between random effects were defined. Additive residual error of 0.2 mg/L is used. Pharmacokinetic sampling is performed at 0.1, 0.25, 0.5, 1, 2, 3, 5, 8 and 12 h after 100 mg study drug dosing.

Data for 3000 subjects were simulated, and models were fitted to datasets of 25, 50, 75, 100, 250, 500, 750, 1000, 2000 and 3000 subjects. Estimation methods were FO-aggregate, FO-individual, FOCE-aggregate, FOCE-individual, MC-aggregate and SAEM-individual. For FOCE-aggregate and MC-aggregate methods, the number of Monte Carlo generated random effect values was either 300 or the number of subjects in the dataset, whichever was higher.

To verify that the minimum number of 300 Monte Carlo generated random effect vectors was adequate, the Monte Carlo approximation standard error of the log-likelihood was calculated via leave-one-out cross-validation. Briefly, each of the 300 random effects vectors was sequentially left out of the log-likelihood calculation, resulting in 300 leave-one-out log-likelihood values. The standard deviation of these 300 log-likelihood values was calculated to get the Monte Carlo approximation error of log-likelihood.

### Case 3: optimal design

This case example shows that aggregate-data and individual-data FO approximations result in identical predicted relative standard errors. It also explores differences in relative standard errors predicted by aggregate-data FOCE versus individual-data FOCE approximation, and standard errors predicted by aggregate-data MC versus Monte Carlo approximation [14]. The model defined in Case 2 was used, and the number of subjects was set to 100. Expected aggregate data were simulated with FO, FOCE and MC approximations of aggregate data (equations 15–18). Then, the Hessian of aggregate data log-likelihood as a function of parameters was calculated using each of the approximations. The consistency of the expected standard errors calculated this way were compared with the expected standard errors calculated with published expressions of population FIM. The FO and FOCE approximated population FIM was calculated with the established R library *PopED*, with 300 random effect Monte Carlo samples for the FOCE approximation. The Monte Carlo approximated population FIM, as detailed by Riviere et al. [14], was calculated with the R library *MIXFIM* with 5000 MC samples and 500 MCMC samples.

### Case 4: stochastic simulation and estimation

This case example demonstrates how the aggregate-data expressions can be used to replace stochastic simulations and estimations. The data-generating model was defined as a transit compartmental model with mean transit time of 1 h and 2 transit compartments, both having log-normally distributed inter-individual variability with log-standard deviation of 0.3. The data-analytic model remained the same as defined in Case 2. The number of subjects was set to 100. Expected aggregate data were simulated using equations 17–18. The data-analytic model was fitted to the expected aggregate data using aggregate-data FO, FOCE and MC methods, and parameter estimates together with expected standard errors were calculated for each method. For conciseness, this procedure is from now on referred as “aggregate-data OD”. In this case, the data-analytic model was different from the data-generating model and therefore it would not have been possible to use the previously published optimal design expressions.

The standard errors, predicted by the aggregate-data estimation, were evaluated by comparing them to SSE results. A total of 200 datasets were first simulated as individual data using the data-generating model, and the data-analytic model was fitted to each of the datasets using the individual-data FO, FOCE and SAEM algorithms; this procedure is henceforth referred as “individual-data SSE”. Then, from each of the generated datasets, an aggregate dataset was calculated using equations 7 and 8 and the data-analytic model was fitted using the aggregate-data FO, FOCE and MC methods; this procedure is henceforth referred as “aggregate-data SSE”. The means and standard deviations of the parameter estimates were calculated for both individual-data FOCE estimations and aggregate-data FOCE estimations.

### Software and algorithms

R version 3.6.0 was used for computations and visualizing the results. For numerical integration across random effect values, Sobol-sequenced random numbers were used via *randtoolbox* R library, because these low discrepancy sequences are superior to pseudo-random numbers for numerical integration [15]. The *nlmixr* R library was used for fitting the FO, FOCE and SAEM models to individual-level data [16]. The *PopED* R library was used for generating the expected FIM for FO and FOCE approximations [17]. Although *PopED* uses different expressions for population FIM than the one featured in Appendix 1 [12], the two sets of expressions yield identical results [18] and thus the use of *PopED* is justified. The *MIXFIM* R library was used to calculate the Monte Carlo exact population FIM [14]. The R library *numDeriv* was used for calculation of accurate numerical derivatives. In addition, several other R libraries (*tidyverse*, *Rcpp*) were used to speed up the computations and to keep the code concise. The source code for all computations is available as an electronic supplement.

## Results

### Case 1: log-likelihood value comparison

This case example demonstrates that the individual-data and aggregate-data FO approximations result in identical log-likelihood values. As shown in Table 1 for FO method, there is a perfect match between the OFV values calculated with the aggregate data method versus the OFV values calculated with the individual data method. For FOCE and FOCEI, the two methods give similar but not identical results. This indicates that the FOCE(I) aggregate data method is not equivalent to the FOCE individual data method.

**Table 1:** Objective function values for the Wang 2007 model and dataset [13], comparing the aggregate data estimation method to results from nlmixr and the original published calculations

Approximation	Residual error type	Aggregate data	Reference
**FO**	**Additive**	**0.0258**	**0.0258**
FOCE	Additive	− 0.0659	− 2.0588
**FO**	**Proportional**	**39.2132**	**39.2132**
FOCE	Proportional	39.2008	39.2067
FOCEI	Proportional	39.2027	39.4576

The reference values are those originally reported for NONMEM [13], and subsequently replicated with nlmixr. The rows where the aggregate data estimation matches perfectly the individual data estimation are highlighted in bold

### Case 2: parameter estimation accuracy

**Fig. 1:**
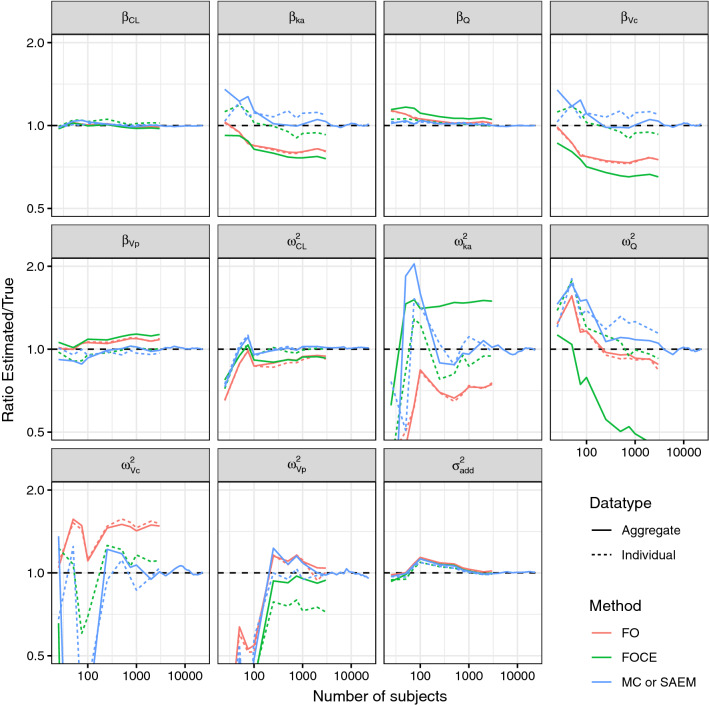
Parameter estimate ratios for different estimation algorithms as a function of number of simulated subjects. The exact likelihood estimation method for individual data was SAEM, and for aggregate data the aggregate-data MC estimation method

Figure 1 shows that the FO method gives nearly identical results for aggregate and individual data. This further confirms that the aggregate data are a sufficient statistic of the individual data in the case of FO method. The FO and FOCE methods, either aggregate or individual-data based, do not converge to the correct parameter values. This is expected, since the FO and FOCE likelihood functions are approximations, not guaranteed to lead to the exactly correct parameter estimates.

The SAEM method converges to the correct parameter estimates roughly when the dataset has 3000 thousand subjects. There seems to be some remaining bias in parameters $\beta_{ka}$, $\beta_{Vc}$ and $\omega_{Q}^{2}$, however it can safely be assumed that this remaining bias would also disappear with further increasing dataset size, as the SAEM has been proven to converge to the exact maximum likelihood estimates [9]. The aggregate-data MC method also converges to the correct parameter estimates albeit slower, in this case with a dataset size up to 23,000 subjects. Again, the actual proof is mathematical (see Appendix 2), and the simulation example shown here serves as a sanity check.

For the aggregate-data MC estimation method with 300 MC simulated subjects, the Monte Carlo approximation standard error of the log-likelihood function was calculated via leave-one-out cross-validation. The approximation standard error was 0.0011 per one subject in the dataset, and would e.g. correspondingly be 1.1 for a dataset of 1000 thousand subjects. This approximation error was considered to be both acceptable, and to have a sufficient safety margin. Thus, it was concluded that 300 MC simulated subjects is an acceptable minimum to use in Case examples 2, 3 and 4.

### Case 3: optimal design

**Fig. 2:**
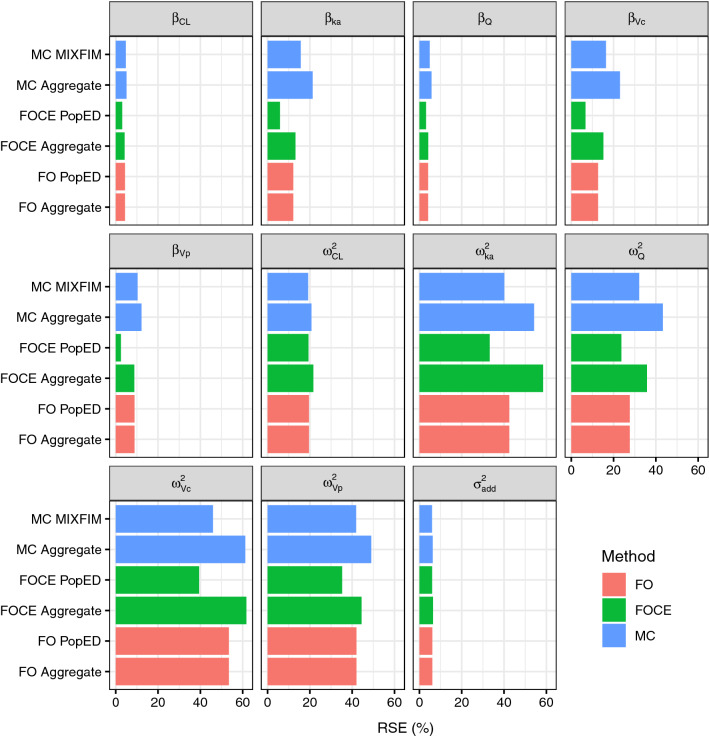
Predicted relative standard errors for a study of 100 subjects on the basis of different optimal design algorithms. RSE% is relative standard error. FO refers to first-order estimation, FOCE refers to first-order conditional estimation, and MC refers Monte Carlo approximation of the log-likelihood

Figure 2 shows that the FO method gives identical results for aggregate and individual data, as expected (see Appendices 1 and 3). The highest relative difference between the two methods was a 0.045% greater RSE predicted by PopED, as compared to the aggregate data log-likelihood, and this difference is likely to result from numerical differences in computations.

For FOCE the method, the RSE predictions differ between PopED and the aggregate data log-likelihood method, as is expected based on the implementation details. In general, the standard errors predicted for the FOCE aggregate method are higher. Similarly, the standard errors predicted by MIXFIM are lower than the standard errors predicted by the MC aggregate method.

The highest relative standard errors are predicted for the random effects variances of Vc, Vp, Q and KA, followed by the fixed-effects estimates of KA and Vc (Fig. 2). These predictions generally agree with the results presented in Fig. 1.

### Case 4: stochastic simulation and estimation

**Fig. 3:**
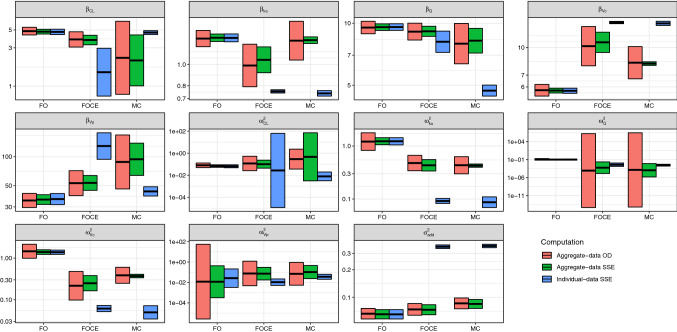
The parameter estimates and their variances when the data-generating model is different from the data-analytic model. The “Aggregate-data OD” refers to fitting the data-analytic model to the expected data, simulated from the data-generating model. The “Aggregate-data SSE” and “Individual-data SSE” labels refer to repeatedly simulating a dataset of 100 individuals and fitting a model to both the simulated individual data, and to aggregate data calculated from the simulated data. FO refers to first-order estimation, FOCE refers to first-order conditional estimation, and MC refers to SAEM algorithm for individual data, and aggregate-data MC method for aggregate data

As seen in Fig. 3, there was an almost perfect match between the aggregate-data OD, aggregate-data SSE and individual-data SSE parameter estimates for FO method. Further, for nearly all parameters there is a good agreement between aggregate-data OD and aggregate-data SSE procedure results, for the non-FO estimation methods. These comparisons show that the aggregate-data OD methods reliably predict the sampling distributions of parameters obtained from aggregate-data SSE.

However, the individual-data SSE procedure gave very differing results for non-FO estimation methods. Generally, random effects variances were lower for individual-data SSE procedure for non-FO estimation methods, and residual variance was higher. The fixed-effects estimates also differed, with no clearly identifiable trends. This demonstrates that when the data-generating model is different from the data-analytic model, the individual-data algorithms and the aggregate-data algorithms do not necessarily converge to the same parameter estimates.

## Discussion

In this paper, we have presented a method for estimation of nonlinear mixed-effects model parameters based on aggregate data of the actual observations. The aggregate data in this paper refers to the mean vectors and the variance–covariance matrix of observations. We have shown how the expressions can be used for fitting models, performing optimal design, and replacing computation-intensive SSE procedures with a faster and more deterministic alternative: Fitting aggregate data models to asymptotically simulated aggregate data. We have shown both mathematically and via computational examples that the aggregate-data FO and individual-data FO algorithms produce identical results given data.

Using FO method, the individual-data and aggregate-data modelling methods are mathematically identical, as was shown in Appendix 1. This means that the parameter estimation properties and optimal design properties are also identical. In the case of FO method, the $\tilde{\user2{y}}$ and $\tilde{\user2{V}}$ are the same for each individual with the same study design and covariate values, and the observed mean vector and variance–covariance matrix are a sufficient statistic for the individual observed data. The implementation presented here is expected to be faster since the log-likelihood for all subjects with an identical design is calculated at once, and not one individual at a time. Therefore, with N subjects, if the individual-data FO log-likelihood evaluation takes x time units, the aggregate-data FO method will take x/N time units plus the time for the computation of the one additional term that is not included in the individual-data log-likelihood (compare Eqs. 10 and 14).

While this manuscript deals with aggregate data, the definition of designs in equations 1 and 2 also allows data from a single individual with a unique design, i.e. unique sampling timepoints. This would subsequently result in individual-level data, because the data could not be grouped with other observations occurring at the same sampling timepoints. If individual data are included as “aggregate data”, then the mean vector becomes the vector of observed data, and the variance–covariance matrix becomes a matrix of zeros. Consequently, the aggregate-data log-likelihood outlined equation 14 would collapse to the individual-data log-likelihood outlined in equation 10. In practice, the aggregate-data FO would then function equivalently to individual-data FO estimation, whereas aggregate-data FOCE and aggregate-data MC estimation methods would function as computationally and statistically inefficient individual-data estimation algorithms.

The individual-data FO and the aggregate-data FO methods gave practically identical results in Case 1 and Case 3, however small differences in parameter estimates were observed in Case 2. We suspect that the small differences between the parameter estimates are caused by differences in implementation of computations. Thus, although the mathematical expressions of individual-data and aggregate-data produce identical results (Appendix 1), small differences can be seen in the computational examples due to differences in numerical implementation.

In Case 4: Stochastic simulation and estimation, we directly fitted the data-analytic model to the expected dataset, given the study design and the data-generating model. The results of this analysis were compared to two SSE scenarios, the first of which was performed based on individual-level data, and the second of which was performed based on aggregate-level data. The mean and variability of parameter estimates from the aggregate-level SSE was almost identical to the parameter estimate sampling distributions predicted by aggregate-data OD. However, the individual-data SSE parameter estimates with FOCE and SAEM algorithms differed from the aggregate-data FOCE and aggregate-data MC parameter estimates. This observation reconfirms that there are differences between the individual-level estimation and aggregate-level estimation algorithms, except for the FO approximation.

For the aggregate-data MC method, the required number of simulated subjects, $N_{sim}$, is dependent on both model and design. A higher number of simulated subjects will likely be required as the number of random effects increases, as the variance of random effects increases, or as the sensitivity of model predictions to random effects increases. Additionally, design aspects such as the number and timing of observations can affect the required number of simulated subjects. An excessively high $N_{sim}$ will result in needlessly slow computation, whereas a too low $N_{sim}$ will result in inaccurate results. In this manuscript, the minimum number of simulated subjects was set to 300, which was empirically found to produce a robust calculation of log-likelihood for the data-analytic model used in Case example 2; for details, see section Results: Case Example 2. As a potential future improvement, it should be possible to dynamically adjust the $N_{sim}$ so that the Monte Carlo approximation error of the log-likelihood function satisfies some constraint.

### Potential applications

Model-based meta-analysis traditionally refers to collecting data from multiple studies, possibly using only literature reports of aggregate data such as means and variances. The methods presented here allow the inclusion of aggregate data in the form of variance–covariance matrices. More so, the methods demonstrated here allow the simulation of aggregate data from literature models, and using these simulated aggregate data as part of fitting the meta-analytic model. In other words, data can be extracted from multiple literature-reported models and combined into a single meta-analytic model. This is an improvement, since thus far it has been necessary to choose one model if multiple models have been reported for a phenomenon.

For using literature models as data, it is not necessary to know the variance–covariance matrix of the model parameters; it is sufficient to only know the model structure, the parameter estimates, and the design aspects such as the PK sampling timepoints. If the literature model is not well-informed, then the lack of information will be captured by the sparsity of the data together with the high variances in the simulated variance–covariance matrix. This was practically demonstrated in Case 3, where only the model and the design were required to compute the expected Fisher information of model parameters, from which the variance–covariance matrix of model parameters can be calculated.

For optimal design, the proposed expressions allow optimizing designs of the data-analytic model when the data-generating model is different from the data-analytic model. In practice, this could mean using a PBPK model as the data-generating model and optimizing the sampling schedule for a two-compartment population PK model. In previous work, there have been examples of a multi-step approach, where data are first simulated from a PBPK model, a population PK model is fitted to the simulated data, and then the population PK model is used as the basis for optimal design [19–21]. The attractive alternative presented here is that a one-step approach can be used, i.e. the “fitting” of the population PK model is performed at the same step as the optimal design.

### Limitations

The aggregate data estimation methods proposed in the current manuscript are based on the mean vector and the variance–covariance matrix of observations. An important limitation to using literature-reported data is that the covariances of observations are rarely reported, typically only means and variances can be extracted from the literature. If no other data than means and variances are available, then it is still theoretically possible to fit some simple nonlinear mixed-effects models, if the random effects affect the variances in a uniquely identifiable way. This scenario would be similar to fitting individual-data nonlinear mixed effects models to datasets with only one observation per subject, which is also theoretically possible. However, in practice it is difficult to make meaningful inferences from such models. To conclude, if only means and variances are available, fitting nonlinear mixed-effects to these data may not be worthwhile. However, aggregate data of means and variances can readily be used jointly with individual data in nonlinear mixed-effects model estimation.

Apart from FO approximation, population modelling with individual data is expected to always be more powerful than modelling with aggregate data. This was demonstrated in the Case 3, in which the expected standard errors for aggregate-data FOCE were higher than those for individual-data FOCE, and likewise the standard errors for aggregate-data MC estimation were higher than those predicted by MIXFIM. The likely reason for this inefficiency of aggregate data estimation is that information is lost when summarized only by means and variance-covariances, i.e. the first and second statistical moments. This amounts to assuming that the data are normally distributed. The third and fourth statistical moments are skewness and kurtosis. Theoretically, implementing skewness and kurtosis as a form of aggregate data would likely further improve the efficiency of aggregate data MC estimation algorithm, however deriving the log-likelihood expressions for skewness and kurtosis would be a challenge.

Case 4 demonstrated that the parameter estimates obtained by aggregate data estimation can be different from parameter estimates obtained by individual data estimation when the data-analytic and the data-generating models are different. Because the data-analytic model is different from the data-generating model, the “correct” parameter estimates for the data-analytic model are unknown. Thus, although Fig. 3 shows that e.g. the residual variance parameter resulting from the aggregate-data estimation was closer to the residual variance value of 0.04 of the data-generating model, this should not be interpreted as aggregate-data estimation being superior to individual-data estimation. It is more relevant to consider the general properties of aggregate-data versus individual-data estimation when deciding which one is more accurate: Whereas the aggregate data estimation methods must assume that the random effects are distributed perfectly normally, individual data estimation methods can allow some degree of skewness or kurtosis in random effects distribution if it results in a better agreement between predictions and data. As such, the aggregate-data estimation methods are likely less robust towards model misspecification than the individual-data estimation methods. Individual-data FOCE and SAEM estimation methods are thus expected to be superior to the aggregate-data FOCE and MC estimation methods in real-life pharmacometric analyses, where the true data-generating model is unknown. However, it is worth noting that even when conducting individual-data modelling, identifying the distribution of random effects as clearly non-normal would likely lead to model refinement until no obvious misspecification can any longer be detected. Furthermore, even if the individual-data estimation methods are more robust against non-normality of the random effects, this advantage is lost when parametrically simulating future trials from the models.

A further reason for individual-data FOCE being superior to aggregate-data FOCE is that when individual data are available, the log-likelihoods are calculated individually and only summed together at the end. On the other hand, when aggregate data are used, the expected mean vector $\tilde{\user2{y}}$ and variance–covariance matrix $\tilde{\user2{V}}$ are calculated based on a set of quasi-random individual parameters (equations 15–16). Then, the mean expected $\tilde{\user2{y}}$ and $\tilde{\user2{V}}$ are used in the calculation of aggregate data log-likelihood. However, the individual-data log-likelihood (equation 10) involves nonlinear functions such as the inverse of $\tilde{\user2{V}}$, and taking a mean of inverse is not the same taking an inverse of mean. Therefore, taking a sum of individual log-likelihoods (calculated based on individually predicted $\tilde{\user2{y}}$ and $\tilde{\user2{V}}$) is more accurate than calculating an aggregate-data log-likelihood on the basis of mean predicted $\tilde{\user2{y}}$ and $\tilde{\user2{V}}$.

For the above reasons, the usefulness of the aggregate-data FOCE approximation is limited. The approximation is computationally expensive because derivatives need to be calculated for a large set of random effects, while there is no guarantee that estimation results would be identical to those estimated by individual-data FOCE method. The aggregate-data MC approximation is generally faster than aggregate-data FOCE approximation due to not having to calculate derivatives. Further, the aggregate-data MC approximation is guaranteed to asymptotically converge to the correct parameter estimates if the data-analytic model is the same as the data-generating model. Meanwhile, the aggregate-data FO method is guaranteed to give results identical to individual-data FO method, regardless of whether the data-analytic model is the same as the data-generating model. To summarize, aggregate-data FO and MC approximations are considered useful, whereas aggregate-data FOCE is not.

The currently proposed expressions do not include covariates, but it should be easy to extend the framework to include them using e.g. the results of Hooker and coworkers [17, 22]. Further, inter-occasion variability is not included in the currently proposed expressions, but should be easy to include in the same manner as done by Retout and colleagues [12]. Indeed, the currently proposed expressions have reserved the subscript *j* exactly for the purpose of denoting *j*th occasion for *i*th individual.

## Conclusions

The presented methods for fitting nonlinear mixed-effects models to aggregate data are considered a valuable addition to the pharmacometric modelling toolbox. Future studies should explore the properties of aggregate-data estimation in model-based meta-analysis, and in conducting optimal design when the data-analytic model is different from the data-generating model.

### Electronic supplementary material

Below is the link to the electronic supplementary material.

Supplementary file1 (R 53 kb)

## Appendix 1: Derivation of aggregate-data log-likelihood from individual-data FO log-likelihood

In this Appendix, the FO log-likelihood expression for aggregate data (equation 14) is derived from the FO log-likelihood expression for individual data (equation 10).

We start with the log-likelihood expression for all data, as the sum of log-likelihoods of individual data. We note that in FO method, $\tilde{\user2{V}}_{i}$ is same for all individuals with the same design factors and can thus be substituted with $\tilde{\user2{V}}$.

$$\begin{gathered} \log \left( {L\left( {{\varvec{y}}{|}{{\varvec{\Psi}}}} \right)} \right) = - \frac{1}{2}\mathop \sum \limits_{i} \left( {tr\left( {{\varvec{R}}_{i} \tilde{\user2{V}}_{i}^{ - 1} } \right) + {\text{log}}\left| {\tilde{\user2{V}}_{i} } \right|} \right) \hfill \\ \,\,\,\,\,\,\,\,\,\,\,\,\,\,\,\,\,\,\,\,\,\,\,\,\,\,\,\,\,\,\,\, = - \frac{1}{2}\left( {tr\left( {\left( {\mathop \sum \limits_{i} {\varvec{R}}_{i} } \right)\tilde{\user2{V}}^{ - 1} }  \right) + N {\text{log}}\left| {\tilde{\user2{V}}} \right|} \right) \hfill \\ \end{gathered}$$

Observing the matrix $\mathop \sum \limits_{i} {\varvec{R}}_{i}$

$$\mathop \sum \limits_{i} {\varvec{R}}_{i} = \mathop \sum \limits_{i} {\varvec{y}}_{res,i}^{T} \times {\varvec{y}}_{res,i}$$

The mth row and nth column of this matrix correspond to

$$\left( {\mathop \sum \limits_{i} {\varvec{R}}_{i} } \right)_{m,n} = \mathop \sum \limits_{i} \left( {y_{im} - \tilde{y}_{m} } \right)\left( {y_{in} - \tilde{y}_{n} } \right)$$

We can twice include the terms for mean observations as follows

$$\left( {\mathop \sum \limits_{i} {\varvec{R}}_{i} } \right)_{m,n} = \mathop \sum \limits_{i} \left( {\overline{y}_{m} + \left( {y_{im} - \overline{y}_{m} } \right) - \tilde{y}_{m} } \right)\left( {\overline{y}_{n} + \left( {y_{in} - \overline{y}_{n} } \right) - \tilde{y}_{n} } \right)$$

Expanding the above gives

$$\left( {\mathop \sum \limits_{i} {\varvec{R}}_{i} } \right)_{m,n} = \mathop \sum \limits_{i} \left( {\begin{array}{*{20}c} { + \overline{y}_{m} \overline{y}_{n} + \overline{y}_{m} \left( {y_{in} - \overline{y}_{n} } \right) - \overline{y}_{m} \tilde{y}_{n} } \\ { + \left( {y_{im} - \overline{y}_{m} } \right)\overline{y}_{n} + \left( {y_{im} - \overline{y}_{m} } \right)\left( {y_{in} - \overline{y}_{n} } \right) - \left( {y_{im} - \overline{y}_{m} } \right)\tilde{y}_{n} } \\ { - \tilde{y}_{m} \overline{y}_{n} - \tilde{y}_{m} \left( {y_{in} - \overline{y}_{n} } \right) + \tilde{y}_{m} \tilde{y}_{n} } \\ \end{array} } \right)$$

It can be noted that $\overline{\user2{y}}$ is not affected by i. Further, $\tilde{\user2{y}}$ is not affected by i. Even though $\left( {y_{ik} - \overline{y}_{k} } \right)$ are different at each value of i, the mean of these values is zero. Therefore,

$$\mathop \sum \limits_{i} \left( {y_{im} - \overline{y}_{m} } \right)\left( {\overline{y}_{n} - \tilde{y}_{n} } \right) = \left( {\overline{y}_{n} - \tilde{y}_{n} } \right)\mathop \sum \limits_{i} \left( {y_{im} - \overline{y}_{m} } \right) = 0$$

$$\mathop \sum \limits_{i} \left( {y_{in} - \overline{y}_{n} } \right)\left( {\overline{y}_{m} - \tilde{y}_{m} } \right) = \left( {\overline{y}_{m} - \tilde{y}_{m} } \right)\mathop \sum \limits_{i} \left( {y_{in} - \overline{y}_{n} } \right) = 0$$

Also, it can be observed that the sum of residual cross-products is actually maximum likelihood variance–covariance matrix:

$$\mathop \sum \limits_{i} \left( {y_{im} - \overline{y}_{m} } \right)\left( {y_{in} - \overline{y}_{n} } \right) = N\frac{{\mathop \sum \nolimits_{i} \left( {y_{im} - \overline{y}_{m} } \right)\left( {y_{in} - \overline{y}_{n} } \right)}}{N} = NV_{mn}$$

Substituting this back to $R_{m,n}$ and removing the terms that amount to zero gives

$$\left( {\mathop \sum \limits_{i} {\varvec{R}}_{i} } \right)_{m,n} = N\left( {V_{mn} + \left( {\overline{y}_{m} - \tilde{y}_{m} )(\overline{y}_{n} - \tilde{y}_{n} } \right)} \right)$$

With this result, we can write

$$\mathop \sum \limits_{i} {\varvec{R}}_{i} = N{\varvec{V}} + N\left( {\overline{\user2{y}} - \tilde{\user2{y}}} \right)^{T} \left( {\overline{\user2{y}} - \tilde{\user2{y}}} \right)$$

Further substituting this into the log-likelihood expression for individual data, and omitting the subscript i, gives

$$  \begin{gathered}   \log \left( {L\left( {\varvec{y}|\varvec{\Psi} } \right)} \right) =  - \frac{1}{2}\left( {tr\left( {N\varvec{V}{\tilde{\user2{V}}}^{{-1}}} \right) + N\,tr\left( {\left( {\overline{\user2{y}} - {\tilde{\varvec{y}}}} \right)^{T} \left( {\overline{\user2{y}}  - {\text{ }}\tilde{\varvec{y}}} \right)\tilde{\user2{V}}^{{ - 1}} } \right) + N~\log \left| {\tilde{\user2{V}}} \right|} \right) \hfill \\   {\text{ }}\quad \quad \quad \quad \quad  =  - \frac{1}{2}N\left( {tr\left( {\varvec{V}\tilde{\user2{V}}^{{ - 1}} } \right) + \left( {\overline{\user2{y}} - \tilde{\varvec{y}}} \right)^{T} {\text{ }}\tilde{\user2{V}}^{{ - 1}} \left( {\overline{\user2{y}}  - \tilde{\varvec{y}}} \right) + \log \left| {\tilde{\user2{V}}} \right|} \right) \hfill \\  \end{gathered}  $$

Which is termed the aggregate data log-likelihood in the current manuscript.

## Appendix 2:Derivation of the general aggregate-data log-likelihood

With a model that only produces aggregate-level data predictions, the log-likelihood for any individual datapoints is

$$\log \left( {L\left( {{\varvec{y}}{|}{{\varvec{\Psi}}}} \right)} \right) = - \frac{1}{2}\mathop \sum \limits_{i} \left( {{\varvec{y}}_{res,i}^{T} \tilde{\user2{V}}^{ - 1} {\varvec{y}}_{res,i} + {\text{log}}\left| {\tilde{\user2{V}}} \right|} \right)$$

where $\tilde{\user2{V}}$ is the population-level variance–covariance matrix, and ${\varvec{y}}_{res,i}$ is the vector of individual residuals, calculated on the basis of population-level mean predictions and individual data.

Using the same steps as outlined in Appendix 1, it is then possible to show that the aggregate-data log-likelihood can be expressed as

$$\log \left( {L\left( {\varvec{y}|\varvec\Psi } \right)} \right) = - \frac{1}{2}N\left( {tr\left( {\varvec{V}{\tilde{\user2{V}}}^{{ - 1}} } \right) + \left( {\overline{\user2{y}} - \tilde{\user2{y}}} \right)^{T} {\tilde{\user2{V}}}^{{ - 1}} \left( {\overline{\user2{y}} - \tilde{\user2{y}}} \right) + \log \left| {\tilde{\user2{V}}} \right|} \right)$$

This is an exact expression for the aggregate-data log-likelihood. Therefore, maximizing the log-likelihood of this expression as a function of ${{\varvec{\Psi}}}$ will result in the exact maximum likelihood estimates with respect to data, similar to the EM individual-data estimation algorithms such as stochastic approximation EM and importance sampling EM algorithms.

## Appendix 3:Derivation of the expected FIM when the data-generating model is the same as the data-analytic model

Retout et al. [23], derived expressions for the expected FIM given a log-likelihood function.

Translating and extending their notation into the format used in the current manuscript and omitting the constant gives

$$\log \left( {L\left( {{\varvec{\varPsi}},{\varvec{y}}} \right)} \right) = \left( {{\varvec{y}} - \tilde{\user2{y}}} \right)^{T} \tilde{\user2{V}}^{ - 1} \left( {{\varvec{y}} - \tilde{\user2{y}}} \right) + {\text{log}}\left| {\tilde{\user2{V}}} \right|$$

The authors [23] used expressions

$$\frac{{\delta \log \left| {\tilde{\user2{V}}} \right|}}{{\delta \lambda_{k} }} = tr\left( {\tilde{\user2{V}}^{ - 1} \frac{{\delta \tilde{\user2{V}}}}{{\delta \lambda_{k} }}} \right)$$

and

$$\frac{{\delta \tilde{\user2{V}}^{ - 1} }}{{\delta \lambda_{k} }} = - \tilde{\user2{V}}^{ - 1} \frac{{\delta \tilde{\user2{V}}}}{{\delta \lambda_{k} }}\tilde{\user2{V}}^{ - 1}$$

To show that

$$E\left( {\frac{{\delta^{2} \left( { - \log \left( {L\left( {{\varvec{\varPsi}},{\varvec{y}}} \right)} \right)} \right)}}{{\delta {\varvec{\beta}}\delta {\varvec{\beta}}^{T} }}} \right) \cong \frac{{\delta f^{T} \left( {{\varvec{\beta}},{\varvec{\xi}}} \right)}}{{\delta {\varvec{\beta}}}}\tilde{\user2{V}}^{ - 1} \frac{{\delta f\left( {{\varvec{\beta}},{\varvec{\xi}}} \right)}}{{\delta {\varvec{\beta}}}}    E\left( {\frac{{\delta^{2} \left( { - \log \left( {L\left( {{\varvec{\varPsi}},{\varvec{y}}} \right)} \right)} \right)}}{{\delta {\varvec{\beta}}\delta \lambda_{k} }}} \right) \cong  0     E\left( {\frac{{\delta^{2} \left( { - \log \left( {L\left( {{\varvec{\varPsi}},{\varvec{y}}} \right)} \right)} \right)}}{{\delta \lambda_{k} \delta \lambda_{j} }}} \right) \cong \frac{1}{2}tr\left( {\tilde{\user2{V}}^{ - 1} \frac{{\delta \tilde{\user2{V}}}}{{\delta \lambda_{j} }}\tilde{\user2{V}}^{ - 1} \frac{{\delta \tilde{\user2{V}}}}{{\delta \lambda_{k} }}} \right)$$

These expressions were derived with the assumption that the fixed effects do not affect the predicted variance, and the random effects do not affect the predicted mean. Later, the assumptions have been relaxed to allow for fixed effects to affect the predicted variance [12]. The only change is that the expression

$$tr\left( {\tilde{\user2{V}}^{ - 1} \frac{{\delta \tilde{\user2{V}}}}{{\delta \lambda_{j} }}\tilde{\user2{V}}^{ - 1} \frac{{\delta \tilde{\user2{V}}}}{{\delta \lambda_{k} }}} \right)$$

becomes more general

$$tr\left( {\tilde{\user2{V}}^{ - 1} \frac{{\delta \tilde{\user2{V}}}}{{\delta \psi_{j} }}\tilde{\user2{V}}^{ - 1} \frac{{\delta \tilde{\user2{V}}}}{{\delta \psi_{k} }}} \right)$$

and enters also the expressions where fixed-effects are present.

We show that the differentiation of the aggregate data log-likelihood function directly results in the expected FIM expressions [12]. We investigate the most general case where fixed effects can affect both predicted means and variances, and also random effects can affect predicted means and variances.

We go through each of the terms of the aggregate data log-likelihood expression (expression 14). We note that a change in parameters can affect the predicted $\tilde{\user2{V}}$ and $\tilde{\user2{y}}$ but not the observed ${\varvec{V}}$ and $\overline{\user2{y}}$, and we note that $\user2{V\tilde{V}}^{ - 1} = I$. We further note that $\tilde{\user2{V}}$ is symmetric, $\tilde{\user2{V}} = \tilde{\user2{V}}^{T}$ and that the matrix multiplication order can be rearranged within the trace operator. Starting with the first term,

$$\frac{{\delta ^{2} tr\left( {\varvec{V}{\tilde{\user2{V}}}^{{ - 1}} } \right)}}{{\delta \psi _{k} \delta \psi _{j} }} = - \frac{\delta }{{\delta \psi _{k} }}tr\left( {\varvec{V}{\tilde{\user2{V}}}^{{ - 1}} \frac{{\delta {\tilde{\user2{V}}}}}{{\delta \psi _{j} }}~{\tilde{\user2{V}}}^{{ - 1}} } \right){\text{ }} = - tr\left( {\varvec{V}{\tilde{\user2{V}}}^{{ - 1}} \frac{{\delta {\tilde{\user2{V}}}}}{{\delta \psi _{k} }}~{\tilde{\user2{V}}}^{{ - 1}} \frac{{\delta {\tilde{\user2{V}}}}}{{\delta \psi _{j} }}{\tilde{\user2{V}}}^{{ - 1}} + {\varvec{V}{\tilde{\user2{V}}}}^{{ - 1}} \frac{{\delta ^{2} {\tilde{\user2{V}}}}}{{\delta \psi _{j} \delta \psi _{k} }}{\tilde{\user2{V}}}^{{ - 1}} - {\varvec{V}{\tilde{\user2{V}}}}^{{ - 1}} \frac{{\delta {\tilde{\user2{V}}}}}{{\delta \psi _{j} }}{\tilde{\user2{V}}}^{{ - 1}} \frac{{\delta {\tilde{\user2{V}}}}}{{\delta \psi _{k} }}~{\tilde{\user2{V}}}^{{ - 1}} } \right){\text{ }} = tr\left( {2{\varvec{V}{\tilde{\user2{V}}}}^{{ - 1}} \frac{{\delta {\tilde{\user2{V}}}}}{{\delta \psi _{k} }}{\tilde{\user2{V}}}^{{ - 1}} \frac{{\delta {\tilde{\user2{V}}}}}{{\delta \psi _{j} }}{\tilde{\user2{V}}}^{{ - 1}} - {\varvec{V}{\tilde{\user2{V}}}}^{{ - 1}} \frac{{\delta ^{2} {\tilde{\user2{V}}}}}{{\delta \psi _{j} \delta \psi _{k} }}{\tilde{\user2{V}}}^{{ - 1}} } \right) = tr\left( {2\frac{{\delta {\tilde{\user2{V}}}}}{{\delta \psi _{k} }}{\tilde{\user2{V}}}^{{ - 1}} \frac{{\delta {\tilde{\user2{V}}}}}{{\delta \psi _{j} }}{\tilde{\user2{V}}}^{{ - 1}} - \frac{{\delta ^{2} {\tilde{\user2{V}}}}}{{\delta \psi _{j} \delta \psi _{k} }}~{\tilde{\user2{V}}}^{{ - 1}} } \right)$$

Next, we note that

$$\frac{{\delta \left( {\overline{\user2{y}} - \tilde{\user2{y}}} \right)}}{{\delta \psi_{j} }} = - \frac{{\delta \tilde{\user2{y}}}}{{\delta \psi_{j} }}$$

Therefore

$$\frac{{\delta ^{2} \left( {\left( {\overline{\user2{y}} - \tilde{\user2{y}}} \right)^{T} \tilde{\user2{V}}^{{ - 1}} \left( {\overline{\user2{y}} - \tilde{\user2{y}}} \right)} \right)}}{{\delta \psi _{j} \delta \psi _{k} }} = \frac{{\delta \left( { - \left( {\frac{{\delta \tilde{\user2{y}}}}{{\delta \psi _{j} }}} \right)^{T} {\text{ }}\tilde{\user2{V}}^{{ - 1}} \left( {\overline{\user2{y}} - \tilde{\user2{y}}} \right) + \left( {\overline{\user2{y}} - \tilde{\user2{y}}} \right)^{T} {\text{ }}\tilde{\user2{V}}^{{ - 1}} \frac{{\delta \tilde{\user2{V}}}}{{\delta \psi _{j} }}\tilde{\user2{V}}^{{ - 1}} \left( {\overline{\user2{y}} - \tilde{\user2{y}}} \right) - \left( {\overline{\user2{y}} - \tilde{\user2{y}}} \right)^{T} {\text{ }}\tilde{\user2{V}}^{{ - 1}} \frac{{\delta \tilde{\user2{y}}}}{{\delta \psi _{j} }}} \right)~}}{{\delta \psi _{k} }}~{\text{ }} = 2\left( {\frac{{\delta \tilde{\user2{y}}}}{{\delta \psi _{j} }}} \right)^{T} {\text{ }}\tilde{\user2{V}}^{{ - 1}} \frac{{\delta \tilde{\user2{y}}}}{{\delta \psi _{k} }} - 2\left( {\bar{y} - \tilde{\user2{y}}} \right)^{T} {\text{ }}\tilde{\user2{V}}^{{ - 1}} \frac{{\delta ^{2} \tilde{\user2{y}}}}{{\delta \psi _{k} \psi _{j} }} = 2\left( {\frac{{\delta \tilde{\user2{y}}}}{{\delta \psi _{j} }}} \right)^{T} {\text{ }}\tilde{\user2{V}}^{{ - 1}} \frac{{\delta \tilde{\user2{y}}}}{{\delta \psi _{k} }}$$

Because of terms including $\left( {\overline{\user2{y}} - \tilde{\user2{y}}} \right) = 0$ canceling out. Finally,

$$\frac{{{\updelta }^{2} \left( {{\text{log}}\left| {\tilde{\user2{V}}} \right|} \right)}}{{\delta \psi_{j} \delta \psi_{k} }} = \frac{{\delta tr\left( {\tilde{\user2{V}}^{ - 1} \frac{{\delta \tilde{\user2{V}}}}{{\delta \psi_{k} }}} \right)}}{{\delta \psi_{j} }} = tr\left( {\tilde{\user2{V}}^{ - 1} \frac{{\delta^{2} \tilde{\user2{V}}}}{{\delta \psi_{k} \delta \psi_{j} }} - \tilde{\user2{V}}^{ - 1} \frac{{\delta \tilde{\user2{V}}}}{{\delta \psi_{j} }}\tilde{\user2{V}}^{ - 1} \frac{{\delta \tilde{\user2{V}}}}{{\delta \psi_{k} }}} \right)$$

Putting all of the parts together,

$$ E\left( {\frac{{\delta ^{2} \left( { - \log \left( {L\left({{{\varvec{\Psi}}}},{\varvec{y}} \right)} \right)} \right)}}{{\delta \psi _{j} \delta \psi _{k} }}} \right) \cong  - \left( { - \frac{1}{2}} \right)\left( {tr\left( {2\frac{{\delta {\tilde{\user2{V}}}}}{{\delta \psi _{k} }}\tilde{\user2{V}}^{{ - 1}} \frac{{\delta {\tilde{\user2{V}}}}}{{\delta \psi _{j} }}~\tilde{\user2{V}}^{{ - 1}}  - \frac{{\delta ^{2} {\tilde{\user2{V}}}}}{{\delta \psi _{j} \delta \psi _{k} }}~\tilde{\user2{V}}^{{ - 1}} } \right) + 2\left( {\frac{{\delta \tilde{\user2{y}}}}{{\delta \psi _{j} }}} \right)^{T} {\text{ }}\tilde{\user2{V}}^{{ - 1}} \frac{{\delta \tilde{\user2{y}}}}{{\delta \psi _{k} }} + tr\left( {\tilde{\user2{V}}^{{ - 1}} \frac{{\delta ^{2} {\tilde{\user2{V}}}}}{{\delta \psi _{k} \delta \psi _{j} }} - \tilde{\user2{V}}^{{ - 1}} \frac{{\delta \tilde{\user2{V}}}}{{\delta \psi _{j} }}\tilde{\user2{V}}^{{ - 1}} \frac{{\delta \tilde{\user2{V}}}}{{\delta \psi _{k} }}} \right)} \right){\text{ }} = \frac{1}{2}\left( {tr\left( {2\frac{{\delta \tilde{\user2{V}}}}{{\delta \psi _{k} }}\tilde{\user2{V}}^{{ - 1}} \frac{{\delta \tilde{\user2{V}}}}{{\delta \psi _{j} }}~\tilde{\user2{V}}^{{ - 1}} } \right) - tr\left( {\frac{{\delta \tilde{\user2{V}}}}{{\delta \psi _{k} }}\tilde{\user2{V}}^{{ - 1}} \frac{{\delta \tilde{\user2{V}}}}{{\delta \psi _{j} }}~\tilde{\user2{V}}^{{ - 1}} } \right) - tr\left( {\frac{{\delta ^{2} \tilde{\user2{V}}}}{{\delta \psi _{j} \delta \psi _{k} }}~\tilde{\user2{V}}^{{ - 1}} } \right) + tr\left( {\frac{{\delta ^{2} \tilde{\user2{V}}}}{{\delta \psi _{j} \delta \psi _{k} }}\tilde{\user2{V}}^{{ - 1}} } \right) + 2\left( {\frac{{\delta \tilde{\user2{y}}}}{{\delta \psi _{j} }}} \right)^{T} {\text{ }}\tilde{\user2{V}}^{{ - 1}} \frac{{\delta \tilde{\user2{y}}}}{{\delta \psi _{k} }}} \right){\text{ }} = \frac{1}{2}tr\left( {\frac{{\delta {\tilde{\user2{V}}}}}{{\delta \psi _{k} }}\tilde{\user2{V}}^{{ - 1}} \frac{{\delta {\tilde{\user2{V}}}}}{{\delta \psi _{j} }}\tilde{\user2{V}}^{{ - 1}} } \right) + \left( {\frac{{\delta \tilde{\user2{y}}}}{{\delta \psi _{j} }}} \right)^{T} {\text{ }}\tilde{\user2{V}}^{{ - 1}} \frac{{\delta \tilde{\user2{y}}}}{{\delta \psi _{k} }} $$

Finally, with these identities we can conclude that under the assumptions of FO approximation, in which the random effects are not able to affect mean predictions,

$$E\left( {\frac{{\delta^{2} \left( { - \log \left( {L\left( {{\varvec{\varPsi}},{\varvec{y}}} \right)} \right)} \right)}}{{\delta \beta_{j} \delta \beta_{k} }}} \right) \cong \left( {\frac{{\delta \tilde{\user2{y}}}}{{\delta \beta_{j} }}} \right)^{T} \tilde{\user2{V}}^{ - 1} \frac{{\delta \tilde{\user2{y}}}}{{\delta \beta_{k} }} + \frac{1}{2}tr\left( {\tilde{\user2{V}}^{ - 1} \frac{{\delta \tilde{\user2{V}}}}{{\delta \beta_{j} }}\tilde{\user2{V}}^{ - 1} \frac{{\delta \tilde{\user2{V}}}}{{\delta \beta_{k} }} } \right)  E\left( {\frac{{\delta^{2} \left( { - \log \left( {L\left( {{\varvec{\varPsi}},{\varvec{y}}} \right)} \right)} \right)}}{{\delta \beta_{j} \delta \lambda_{k} }}} \right)  \cong \frac{1}{2}tr\left( {\tilde{\user2{V}}^{ - 1} \frac{{\delta \tilde{\user2{V}}}}{{\delta \beta_{j} }} \tilde{\user2{V}}^{ - 1} \frac{{\delta \tilde{\user2{V}}}}{{\delta \lambda_{k} }}} \right)  E\left( {\frac{{\delta^{2} \left( { - \log \left( {L\left( {{\varvec{\varPsi}},{\varvec{y}}} \right)} \right)} \right)}}{{\delta \lambda_{j} \delta \lambda_{k} }}} \right) \cong \frac{1}{2}tr\left( {\tilde{\user2{V}}^{ - 1} \frac{{\delta \tilde{\user2{V}}}}{{\delta \lambda_{j} }}\tilde{\user2{V}}^{ - 1} \frac{{\delta \tilde{\user2{V}}}}{{\delta \lambda_{k} }}} \right)$$

Which is identical to the results derived by Retout et al. [12]. To summarize, in this Appendix we have derived the previously published expressions for expected population FIM using only the expected aggregate data. This approach has pedagogical value because it may be more intuitive for some people than deriving the expected population FIM from individual-data log-likelihood expressions.
